# Case Report: Anti-TNF Treatment Failure in a Patient With Immune Checkpoint Inhibitor-Induced Severe Colitis

**DOI:** 10.3389/fonc.2022.925964

**Published:** 2022-06-23

**Authors:** Leilei Fang, Changqin Liu, Xiaomin Sun, Zhanju Liu

**Affiliations:** Center for IBD Research, The Shanghai Tenth People’s Hospital, School of Medicine, Tongji University, Shanghai, China

**Keywords:** anti-tumor necrosis factor-α, colitis, durvalumab, immune checkpoint inhibitor, programmed cell death ligand 1, vedolizumab

## Abstract

Immune checkpoint inhibitor (ICI)-induced colitis is one of the known complications of therapies targeting cytotoxic programmed cell death protein 1 (PD-1), cytotoxic T lymphocyte-associated antigen 4 (CTLA-4), and programmed cell death ligand 1 (PD-L1). ICI-associated colitis is routinely treated with immunosuppressive therapy, including corticosteroids and/or agents targeting tumor necrosis factor-α (TNF-α). In this report, a 69-year-old male patient developed severe ICI-induced colitis 2 weeks after anti-PD-L1 mAb (i.e., durvalumab) treatment; unexpectedly failed to respond to systemic corticosteroid, anti-TNF, and anti-integrin agents; and unfortunately died in 1 month. This case reminds clinical physicians to be on the alert for early-onset acute ICI-induced colitis and emphasizes that urgent optimized rescue measures are required for patients with severe ICI-induced colitis.

## Introduction

Immune checkpoint inhibitors (ICIs) have shown to be revolutionary in the treatment of various tumors and improve the long-term survival of patients across numerous cancer types (1). ICIs target cytotoxic T lymphocyte-associated antigen 4 (CTLA-4), programmed cell death protein 1 (PD-1), and programmed cell death ligand 1 (PD-L1) and are associated with increased T-cell activation, thereby inducing effective antitumor immune responses in a subset of patients (2, 3). However, ICI therapy may trigger various organ-specific immune-related adverse effects in some patients. Increasing lines of evidence have shown that anti-CTLA-4 therapy (e.g., ipilimumab) usually causes hypophysitis, whereas pneumonitis and thyroiditis appear to be more common with anti-PD-1 therapy (e.g., nivolumab and pembrolizumab) (4). ICIs can lead to gastrointestinal toxicity such as diarrhea (27%–54% with anti-CTLA-4 treatment) and colitis (8%–22% with anti-CTLA-4 treatment and only 1%–2% with anti-PD-1 treatment) (4–7). Very few data are available about gastrointestinal adverse effects associated with anti-PD-L1 agents.

ICI-associated colitis is routinely treated with immunosuppressive therapy, including corticosteroids and/or agents targeting tumor necrosis factor-α (TNF-α) or integrin α4β7 (8). Here, we described that a 69-year-old male patient presented severe ICI-induced colitis due to anti-PD-L1 mAb (i.e., durvalumab) treatment for small cell lung cancer (SCLC; poorly differentiated neuroendocrine carcinoma) but failed to respond to rescuable therapy including systemic corticosteroids, anti-TNF (i.e., infliximab), and anti-integrin (i.e., vedolizumab) and unfortunately died of massive bleeding, which may contribute to optimizing current treatment strategies for patients with severe ICI-induced colitis.

## Case Presentation

**Figure 1:**
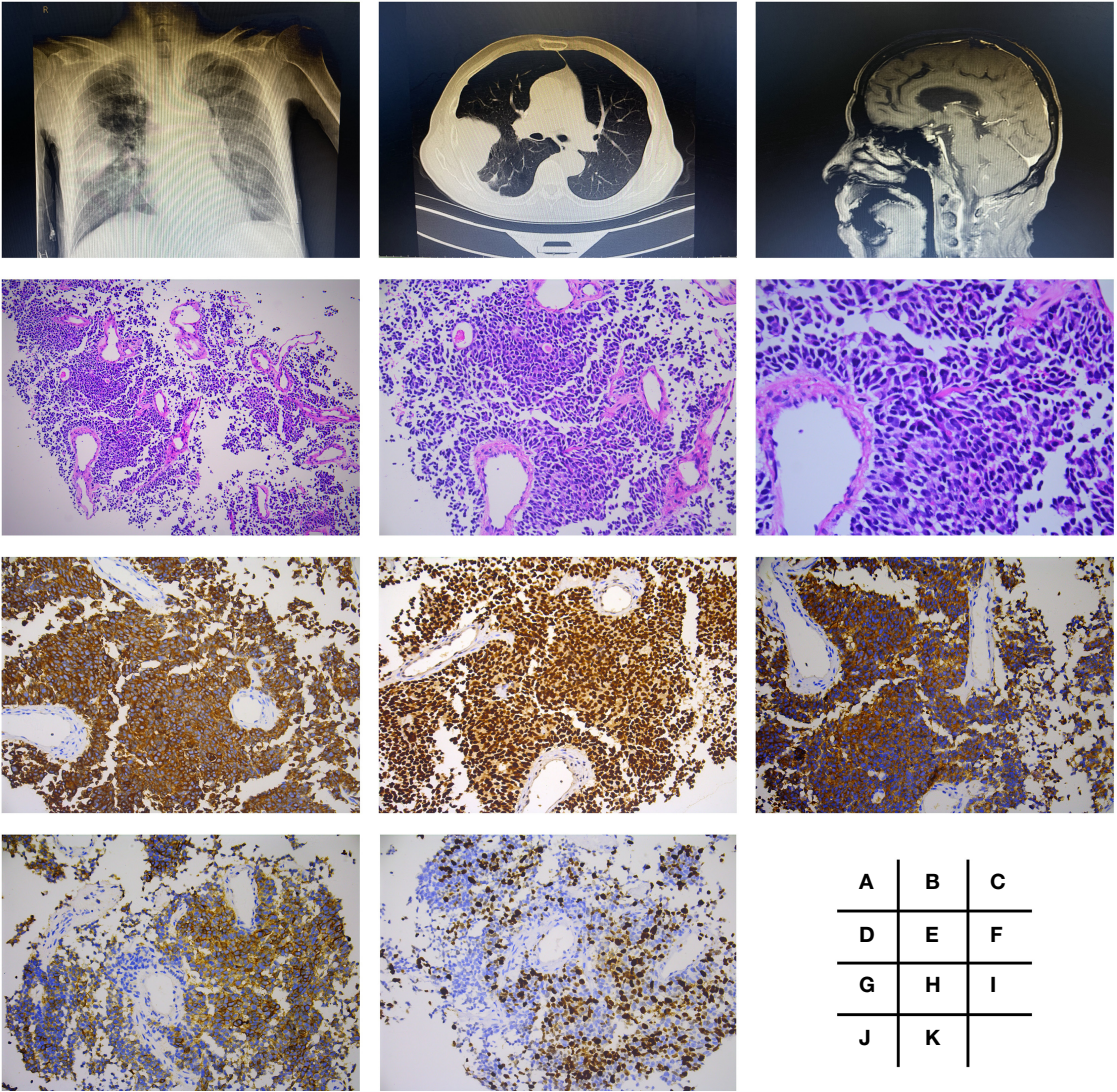
Radiologic findings and histological features of small cell lung cancer (poorly differentiated neuroendocrine carcinoma). Chest X-ray examination showed a mass of high density in right middle lobe **(A)**, which was presented more clearly by CT **(B)**. Brain MRI confirmed metastasis in cerebellar tonsillar **(C)**. H&E staining performed is shown in panels **(D–F)**, which were magnified by ×100, ×200, and ×400, respectively. Further immunohistochemistry staining showed CK7(+) **(G)**, TIF-1(+) **(H)**, SYN(+) **(I)**, CD56(+) **(J)**, and ki-67 (60% +) **(K)**, all with magnification of ×200.

A 69-year-old man with a 1-month history of right chest pain, cough, and expectoration had undergone an X-ray of the chest at the outpatient clinic of the Shanghai Tenth People’s Hospital of Tongji University (Shanghai, China) on October 23, 2021. Chest X-ray examination showed a high-density mass in the middle lobe of the right lung (**Figure 1A**). Further CT-guided percutaneous lung biopsy of these lung nodules was performed on November 10, 2021. One week later, biopsy pathology revealed CK7(+), TIF-1(+), SYN(+), CD56(+), and ki-67 (60% +), confirming that it was poorly differentiated neuroendocrine carcinoma (SCLC) (**Figures 1B, D–K**). What was worse, PET-CT showed that he had pleural, mediastinal lymph node, and cerebellar metastases. Brain MRI confirmed metastasis of lung cancer in the cerebellar tonsillar (**Figure 1C**).

Thus, the patient was admitted to the Department of Oncology in our hospital on November 15, 2021. He was a non-drinker and non-smoker, and had tuberculosis 50 years ago, which had been cured already. In addition, he had no history of any other diseases like gastrointestinal diseases and autoimmune diseases or medication use such as non-steroidal anti-inflammatory drugs (NSAIDs), proton pump inhibitors, or probiotics. He also denied any family history of hypertension, diabetes, and tumor. On admission, he had experienced a weight loss of 5 kg in 4 months and gradually worsening right chest pain, cough, and expectoration despite that he had taken an antibiotic (i.e., amoxil capsule) since 1 week ago. His Karnofsky performance score (KPS) was 80, and his body mass index (BMI) was 19.37. Physical examination was almost negative except for mild coarse breath sounds of both lungs on auscultation. Laboratory tests revealed significant increases in carcinoembryonic antigen (CEA; 7.90 ng/ml; n.v., <5.2 ng/ml), carbohydrate antigen (CA)-153 (27.10 U/ml, n.v., <26.4 U/ml), CA-125 (104 U/ml; n.v., <35 U/ml), and neuron-specific enolase (NSE; 32 ng/ml; n.v., <16.3 ng/ml); but C-reactive protein (CRP; 3.30 mg/L; normal value (n.v.), <8.2 mg/L), white blood cell (WBC; 8.56 × 10(9)/L; n.v., 3.5–9.5 × 10^9^/L), red blood cell (RBC, 4.29 × 10^12^/L; n.v., 4.3–5.8 × 10^12^/L), platelet (PLT; 439 × 10^9^/L; n.v., 125–350 × 10^9^/L), and hemoglobin (Hb; 138 g/L; n.v., 130–175 g/L), serum albumin (ALB, 37.3 g/L; n.v., 35–50 g/L), hepatic and renal functions, bleeding time test, D-dimer (0.50 mg/L; n.v., <0.55 mg/L FEU), and fecal tests were almost normal (**Table 1**). Considering that his KPS and BMI are relatively low within normal limits, his oncologist recommended a regimen of anti-PD-L1+IP, namely, durvalumab, a PD-L1 antibody, intravenous injection on the 1st day and irinotecan and cisplatin intravenous injection on the 1st and 8th days, allowing for the evaluation of his tolerance to chemotherapy after the first injection. After informed consent was acquired, durvalumab (1,500 mg; Imfinzi; AstraZeneca, Cambridge, UK) was administered to the patient together with irinotecan (115 mg, Pfizer, Manhattan, NY, USA) and cisplatin (50 mg, Teva Pharma AG, Basel, Switzerland) on November 16, 2021 (1st day). No adverse reaction was reported by him in the next 1 week. No obvious change was found in his laboratory test 1 week later, except for a slight increase of D-dimer (0.95 mg/L) (**Table 1**). Therefore, he received irinotecan and cisplatin of the same dosage as mentioned above on November 23, 2021 (8th day). Later, he presented mild diarrhea (2–3 times/day, grade I) for three days. His oncologist considered it was chemotherapy-induced diarrhea and prescribed him antidiarrheal medicine in an outpatient clinic. However, he developed moderate diarrhea 5–6 times/day and mild abdominal pain (grade II) afterward, even though he had taken antidiarrheal medicine daily at home. Thus, he had to be hospitalized again in the Department of Oncology in our hospital on November 29, 2021. In this instance, laboratory tests revealed significant increases in CRP (59.77 mg/L). As shown in **Table 1**, ALB (33.1 g/L), WBC (1.76 × 10^9^/L), RBC (3.98 × 10^12^/L), and Hb (121 g/L) were significantly decreased. Routine fecal test (RFT) and fecal occult blood test (FOBT) were still negative, as well as fecal ova/parasites tests, stool pathogen cultures, and *Clostridium difficile* toxin tests. In addition, serum coagulation function tests were revealed to be normal, but D-dimer increased significantly (>20.3 mg/L). Further CT angiography excluded mesenteric embolism or bowel perforation.

**Table 1:** Laboratory test results of the baseline and follow-up of the patient.

	2021.11.15	2021.11.22	2021.11.29	2021.12.04	2021.12.08	2021.12.11
CRP	3.3	3.2	59.77	39.74	16.9	191
WBC	8.56	5.21	1.76	5.46*	10.43*	9.69*
RBC	4.29	4.38	3.98	3.97	3.15	1.54
PLT	439	428	346	298	204	302
Hb	138	140	125	124	100	49
ALB	37.3	40.2	33.1	20.4	23.9	18.9
ALT	20.4	15.2	13.6	19.5	17.4	22.7
Cr	67.2	65	89	227.1	91	80
FOBT	–	–	–	++	++++	++++
PT	11.1	10.9	11.2	11.7	12.9	12.9
APTT	29.8	29.7	30.1	34.5	35.9	39.1
D-dimer	0.5	0.95	>20.3	12.82	17.2	8.19

CRP, C-reactive protein (mg/L), n.v., <8.2 mg/L; WBC, white blood cell (×10^9^/L), n.v., 3.5–9.5 × 10^9^/L; RBC, red blood cell (×10_12_/L), n.v., 4.3–5.8 × 10^12^/L; PLT, platelet (×10^9^/L), n.v., 125–350 × 10^9^/L; Hb, hemoglobin (g/L), n.v., 130–175 g/L; ALB, albumin (g/L), n.v., 35–50 g/L; ALT, alanine transaminase (U/L), n.v., <72 U/L; Cr, serum creatinine (mmol/L), n.v., 58–110 mmol/L; FOBT, fecal occult blood test; PT, prothrombin time (s), n.v., 10.8 ± 3 s; APTT, activated partial thromboplastin time (s), n.v., 30.8 ± 10 s; D-dimer, mg/L, n.v., <0.55 mg/L FEU. *After recombinant human granulocyte-stimulating factor injection.

**Figure 2:**
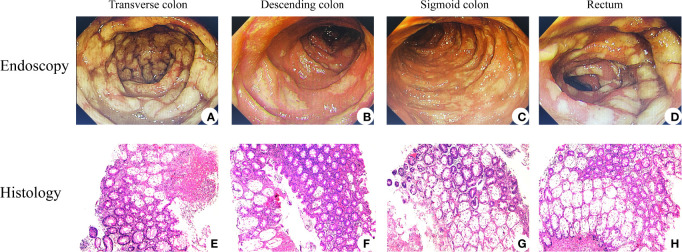
Endoscopic and histological features of immune checkpoint inhibitor (ICI)-related colitis. Endoscopic images of colons revealed that mucosal congestion, edema, rough, unclear perivascular texture, multiple superficial ulcers with purulent secretions, and white moss existed among transverse colon **(A)**, descending colon **(B)**, sigmoid colon **(C)**, and rectum **(D)**. H&E staining performed is shown in panels **(E–H)**, all with magnification of ×100. Histology images courtesy of Qiongyi Huang.

**Figure 3:**
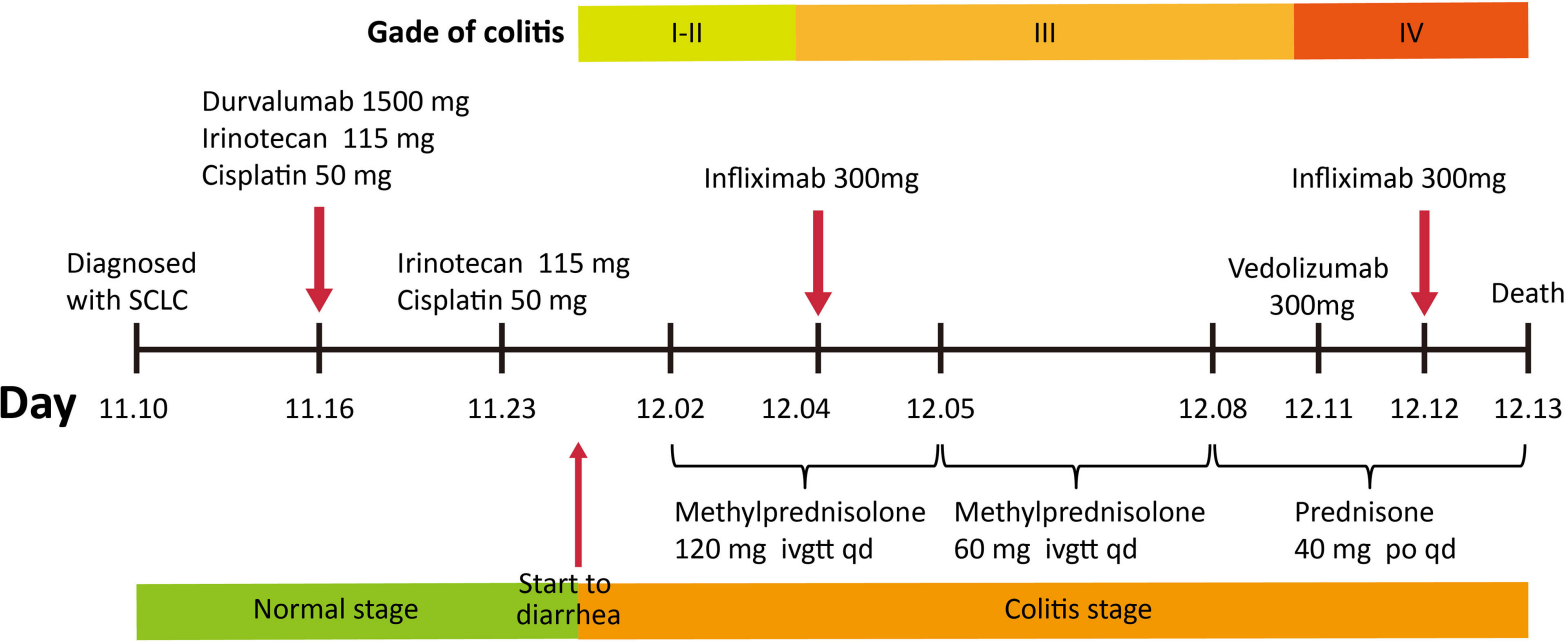
Timeline of medications administered to the patient. SCLC, small cell lung cancer.

His symptoms worsened with bloody diarrhea (6–10 times/day) and moderate abdominal pain (grade III). FOBT became positive (++), but no RBC or WBC was found in RFT. In order to obtain special therapy, he was transferred to the Department of Gastroenterology on December 1, 2021. No evidence of vasculitis, fungal elements, tuberculosis, viral staining for cytomegalovirus, Epstein–Barr virus (EBV)- encoded RNA (EBER), adenovirus, or parasitic infection was found in further laboratory tests. Therefore, the patient was diagnosed with ICI-induced colitis and started on 120 mg of intravenous methylprednisolone daily on December 2, 2021. Three days later, he developed more severe bloody diarrhea (>15 times/day) and moderate-to-severe abdominal pain (grade III), and his ALB decreased significantly. Subsequent colonoscopy showed severe mucosal inflammation from the transverse colon to the anus with multiple ulcerations attached with purulent secretions and moss, consistent with Mayo endoscopic subscore of 3 (**Figures 2A–D**). No obvious abnormality was found in the gastroscope. Histopathological analysis of colon biopsies revealed that there was multiple purulent exudation and necrosis, and colonic crypt atrophy or rupture, suggestive of acute enteritis (**Figures 2E–H**). Considering that he was steroid refractory, 5 mg/kg of anti-TNF mAb (i.e., infliximab; Cilag AG, Schaffhausen, Switzerland) was administered to him on December 4, 2021, followed by a reduced dose of methylprednisolone (60 mg/day) intravenously daily for another 4 days. Meanwhile, enteral nutrition, albumin infusion, empiric antibiotic therapy, and oral probiotics were administered to him daily, and hemostatic therapy was administered to him intermittently when needed. Unexpectedly, his bloody diarrhea became more severe (>20 times/day), and abdominal pain worsened. We immediately switched to total parenteral nutrition on December 7, 2021, and 300 mg of anti-integrin mAb (i.e., vedolizumab; Takeda GmbH, Konstanz, Germany) was administered to the patient to strengthen immunosuppression on December 11, 2021 (**Figure 3**). At this time, laboratory findings reflected the patient’s clinical degradation, including CRP 191 mg/L, ALB 18.9 g/L, and Hb 49 g/L, as shown in **Table 1**. He was urgently treated with fluid resuscitation and blood transfusions on December 11 and 12, 2021, respectively. Furthermore, 300 mg of infliximab was administered to him in advance in order to save him from severe colitis. Unfortunately, he died on December 13, 2021, due to severe lower gastrointestinal bleeding and hemorrhagic shock.

## Discussion

Durvalumab acts by blocking PD-L1 and has been approved by the Food and Drug Administration for the management of extensive-stage SCLC in combination with chemotherapy (9). In the current study, we reported a case of a patient with severe colitis induced by durvalumab without substantial improvement after systemic treatment with corticosteroids, anti-TNF (i.e., infliximab), and anti-integrin (i.e., vedolizumab) agents and who unfortunately died. To date, the mechanisms underlying ICI-associated colitis in patients with tumors are unknown. Mechanistically, defects in the suppressive function of regulatory T cells (Treg) contribute to intestinal mucosal inflammation, which is predominately mediated by type 1 helper T cells (Th1) and Th17 cells, resulting in inflammatory bowel diseases (IBDs) (10). In addition to TNF, various kinds of proinflammatory cytokines are profoundly associated with the pathogenesis of IBD, such as IL-17A, IL-21, and IL-23 (11, 12). As a kind of immune-mediated disease that resembles IBD, ICI-associated colitis has shown suppressive function deficiency of Tregs caused by the PD-1/PD-L1 pathway (3, 13). Recent evidence reported a low density of Foxp3^+^ CD4^+^ T cells (Treg) in the inflamed colonic mucosa of patients with ICI-associated colitis. Following treatment, there was a substantial increase in Foxp3^+^ CD4^+^ T cells in these patients (14). Thus, T-cell subtypes like Foxp3^+^ CD4^+^ T cells and related cytokines like IL-17A and TNF may be potential prognostic factors for ICI-associated colitis.

As a common immune-mediated side effect, the morbidity of colitis in patients using anti-PD-(L)1 agents alone (approximately 1.5%) seems much lower than that in patients administered with anti-PD-(L)1 and chemotherapy (approximately 9.8%) (15, 16), which indicates that chemotherapy may play a role in the catalytic mechanisms underlying ICI-associated colitis. It may also be one of the reasons why the patient in this case developed severe colitis in a relatively short time (2 weeks) compared with the median time as reported (1.5–2 months) (15, 17), which requires further clinical trials in the future.

For severe or life-threatening stages of drug-associated colitis, ICI therapy should be interrupted, and permanent discontinuation is recommended for episodes that progress to colitis. Meanwhile, intravenous corticosteroid treatment (i.e., methylprednisolone, 1–2 mg/kg daily) is recommended (18). Steroid-refractory patients (non-response to intravenous corticosteroids after 72 h) require anti-TNF therapy. Multiple lines of evidence have suggested that higher endoscopic severity scores and histologically diagnosed deep ulcerations may be predictive markers for steroid-refractory ICI-related colitis, likely requiring infliximab therapy (19–21). Therefore, infliximab (5 mg/kg) was administered to patients when colonoscopy showed multiple ulcerations with a Mayo endoscopic subscore of 3. Infliximab has been used successfully in this setting with a dosing regimen similar to that used in IBD patients, starting with an initial dose of 5 mg/kg (18, 22). Moreover, emerging evidence illustrates that anti-integrin (e.g., vedolizumab) agents are also an effective option (23, 24). However, neither infliximab nor vedolizumab has successful outcomes in treating ICI-related colitis in the current case. In this instance, for patients with IBD, tofacitinib, ustekinumab, and calcineurin inhibitors could be appropriate remedial measures, but their safety and efficacy are unclear for patients with ICI-related colitis. In addition, fecal microbiota transplant (FMT) has recently been used for ICI-related colitis, and data from a case series of two patients treated with FMT appear encouraging (25). However, it is very early work, and more data are required in this area before this can be recommended. The patient worsened too quickly to have a total colectomy, which may be ascribed to severe malnutrition caused by the tumor and severe bloody diarrhea.

It gives us a good lesson that those with refractory ICI-related colitis may not respond to treatments of steroids combined with infliximab or vedolizumab, which may even endanger the patient’s life. Additionally, FMT may be an emerging option, although more studies are required to establish its efficacy and safety, aside from total colectomy. More urgent optimized rescue measures are required for patients with severe refractory ICI-related colitis.

## Limitation

There were no T-cell subgroup tests, cytokine tests of peripheral blood, fecal calprotectin tests in the baseline, and follow-up of the patient during treatment due to laboratory limits. Infliximab trough levels of the patient during treatment were not assessed due to the patient’s financial problems.

## Data Availability Statement

The original contributions presented in the study are included in the article/supplementary material. Further inquiries can be directed to the corresponding author.

## Ethics Statement

This article does not contain any studies with human participants or animals performed by any of the authors. Informed consent was given by the patient’s son before clinical data collection.

## Author Contributions

LF, CL, XS, and ZL had the original idea for the article and guided the treatment and management of the patient. LF wrote the article and incorporated the comments from ZL. All authors reviewed and approved the final draft of the article.

## Funding

This work was funded by grants from the National Natural Science Foundation of China (81800487, 91942312).

## Conflict of Interest

The authors declare that the research was conducted in the absence of any commercial or financial relationships that could be construed as a potential conflict of interest.

## Publisher’s Note

All claims expressed in this article are solely those of the authors and do not necessarily represent those of their affiliated organizations, or those of the publisher, the editors and the reviewers. Any product that may be evaluated in this article, or claim that may be made by its manufacturer, is not guaranteed or endorsed by the publisher.
